# Wearable Sensing Systems for Multi-Modal Body Fluid Monitoring: Sensing-Combination Strategy, Platform-Integration Mechanism, and Data-Processing Pattern

**DOI:** 10.3390/bios16010046

**Published:** 2026-01-06

**Authors:** Manqi Peng, Yuntong Ning, Jiarui Zhang, Yuhang He, Zigan Xu, Ding Li, Yi Yang, Tian-Ling Ren

**Affiliations:** 1 School of Integrated Circuit, Tsinghua University, Beijing 100084, China; pmq22@mails.tsinghua.edu.cn (M.P.); ningyt23@mails.tsinghua.edu.cn (Y.N.); zhang-jr23@mails.tsinghua.edu.cn (J.Z.); yh-he23@mails.tsinghua.edu.cn (Y.H.); xuzg24@mails.tsinghua.edu.cn (Z.X.); liding21@mails.tsinghua.edu.cn (D.L.); 2 Beijing National Research Center for Information Science and Technology (BNRist), Tsinghua University, Beijing 100084, China; 3 Center for Flexible Electronics Technology, Tsinghua University, Beijing 100084, China

**Keywords:** body fluid, multi-modal, biochemical sensing, wearable, health monitor, integrated system

## Abstract

Wearable multi-modal body fluid monitoring enables continuous, non-invasive, and context-aware assessment of human physiology. By integrating biochemical and physical information across multiple modalities, wearable systems overcome the limitations of single-marker sensing and provide a more holistic view of dynamic health states. This review offers a system-level overview of recent advances in multi-modal body fluid monitoring, structured into three hierarchical dimensions. We first examine sensing-combination strategies such as multi-marker analysis within single fluids, coupling biochemical signals with bioelectrical, mechanical, or thermal parameters, and emerging multi-fluid acquisition to improve analytical accuracy and physiological relevance. Next, we discuss platform-integration mechanisms based on biochemical, physical, and hybrid sensing principles, along with monolithic and modular architectures enabled by flexible electronics, microfluidics, microneedles, and smart textiles. Finally, the data-processing patterns are analyzed, involving cross-modal calibration, machine learning inference, and multi-level data fusion to enhance data reliability and support personalized and predictive healthcare. Beyond summarizing technical advances, this review establishes a comprehensive framework that moves beyond isolated signal acquisition or simple metric aggregation toward holistic physiological interpretation. It guides the development of next-generation wearable multi-modal body fluid monitoring systems that overcome the challenges of high integration, miniaturization, and personalized medical applications.

## 1. Introduction

Human body fluids, including sweat, saliva, urine, tears, and interstitial fluid (ISF) [1], contain abundant key biomarkers such as electrolytes, hormones, and metabolic products [2,3]. Analyzing these biomarkers can provide comprehensive reflections of the body’s physiological and pathological states, offering valuable insights for monitoring nutrition [4,5,6], metabolism [7,8,9], and hormone levels [10,11,12].

However, conventional monitoring approaches that rely on unimodal information are inherently limited, failing to deliver a holistic assessment of health status [13,14,15]. For instance, in widely adopted biophysical devices like smartwatches, the inability to perform biomolecular analysis restricts the precision and breadth of physiological status assessment [16,17,18]. Therefore, multi-modal monitoring, which combines multiple indicators from body fluids or integrates other physiological signals, has emerged as a promising and pivotal approach to overcome these limitations [6,19,20]. This method enables a more accurate and in-depth representation of physiological conditions.

Wearable sensors represent an emerging platform for continuous, non-invasive, or minimally invasive health and disease monitoring [21], characterized by lightweight design, low power consumption, and portability. These devices are capable of real-time acquisition of physiological data, finding applications in health monitoring, disease prevention, and status recognition. Current multi-modal wearable sensors can integrate chemical, physical, and thermal sensing functions to simultaneously collect and analyze biochemical signals (e.g., electrolytes, metabolites in body fluids) and physical signals (e.g., bioelectricity, temperature, pulse waveform) [3,19,22]. This multi-modal integration provides a promising approach for portable, real-time body fluid monitoring. Furthermore, integrating these wearable sensors with data processing enables the construction of a wearable system that provides comprehensive and personalized monitoring, while supporting user interaction and health prediction [21,23].

**Figure 1:**
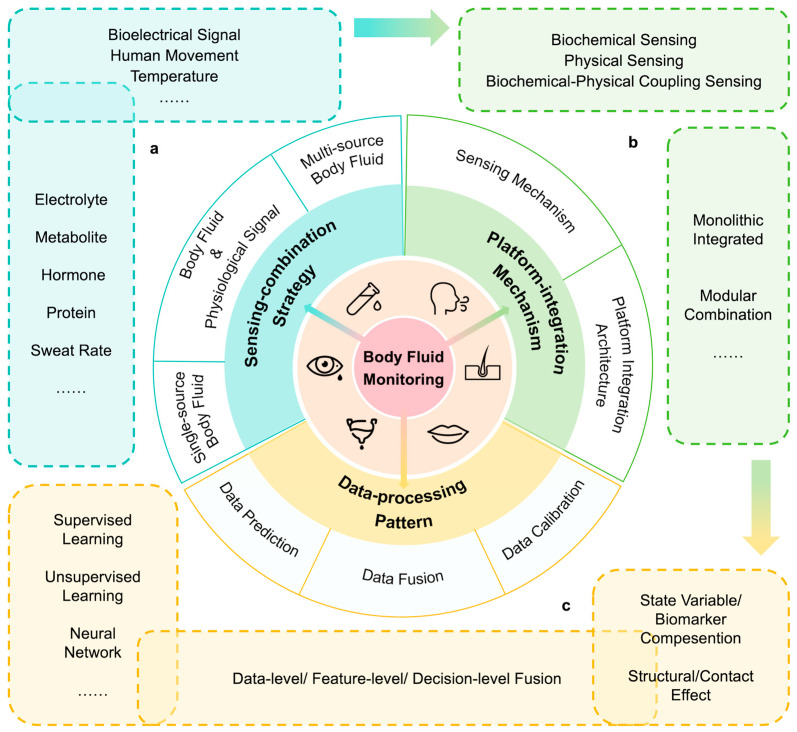
Overview of wearable sensing systems for multi-modal body fluid monitoring. (**a**) Multiple body fluid biomarkers and physiological signals acquired via sensing-combination strategies. (**b**) Response mechanisms of sensors and integration methods of platforms. (**c**) Specific methods and their interplay for data calibration, data prediction, and data fusion.

Within this context, the review aims to provide a systematic overview of the system-level architecture and cutting-edge research in multi-modal body fluid monitoring. Several reviews on body fluid monitoring have been dedicated to single-parameter detection or device-level sensing strategies [24,25,26,27], data-processing methods [28,29,30,31,32], and application optimizations [21,33,34]. We further examine the principles and significance of multi-modal sensing combinations, focusing on the integration of at least two modalities, including biochemical signals, physical signals, or different body fluids. From the system level, we elaborate on the selection, collection, and processing of multi-modal sensing data. Here our discussion is structured around three key dimensions: (i) sensing-combination strategies, including common multi-modal selection and acquisition approaches for physiological signals and their roles in health monitoring; (ii) platform-integration mechanisms, outlining the response mechanisms of various sensor types and the foundational architecture of integrated monitoring systems; and (iii) data-processing patterns, encompassing the analytical frameworks and processing methods applied to multi-modal data streams (Figure 1). Through this systematic overview, the review aims to provide a comprehensive research framework for the development of next-generation portable and wearable multi-modal body fluid monitoring systems, thereby accelerating their transition to personalized healthcare and clinical applications.

## 2. Sensing-Combination Strategy

Multi-modal body fluid information enables a more accurate and comprehensive analysis of the human physiological state. Among many biomarkers and signals, the selection and combination of appropriate acquisition targets can greatly improve the efficiency and reliability of the sensor system. At present, the combination strategy of multi-modal body fluid sensing can mainly be divided into single-source body fluid acquisition, body fluid with other physiological signal-coupled acquisition, and multi-source body fluid acquisition. The sensing range involves a variety of body fluids, and integrates multiple types of physiological signals such as bioelectrical signals, human motion information, and cardiovascular parameters to jointly build a multi-dimensional monitoring system. This section systematically demonstrates how multi-modal humoral sensing of these three strategies has shown a wide range of application prospects in disease diagnosis, sports science, health management, and other fields through the fusion of information from different dimensions.

### 2.1. Single-Source Body Fluid Collection

Sweat, saliva, tears, and interstitial fluid are common detection objects in wearable body fluid sensing. The detection of multiple biomarkers can be achieved by using a single-solution source, so it is also widely used in biomonitoring systems (Table 1).

#### 2.1.1. Sweat

Sweat has become one of the most widely studied body fluids in the field of wearable sensing due to its easy access, rich physiological information, and completely non-invasive monitoring. Sweat contains electrolytes (such as Na^+^, K^+^, Mg^2+^), metabolites (such as glucose, lactic acid, uric acid), hormones, proteins, and other biomarkers, which can reflect the body’s physical health, psychological conditions, and various disease states.

In multi-modal sweat sensing, the monitoring of sweat rate is an important basis for accurate analysis. Sweat rate itself is a key physiological indicator that reflects the body’s hydration and thermoregulation status [47,48]. More importantly, the rate of sweat secretion directly affects the total amount of analytes that can be captured by the sensor per unit time, as well as the concentration of the analyte [49]. Therefore, correlating and normalizing analyte content with real-time sweat rate is crucial for obtaining accurate and reproducible physiological information, which can effectively reduce differences between individuals and under different exercise states, and improve data reliability. The multi-modal biochip developed by Zhong, B. et al. can normalize phenylalanine indicators in sweat by quantifying sweat rate, phenylalanine, and chloride concentrations at the same time [9]. In addition, cross-validation of sweat rate data can be achieved by additionally detecting the concentration of chloride or sodium ions in sweat, improving reliability [9,35].

The combined detection of glucose and cortisol in sweat can effectively reflect the nutritional metabolism of the human body. As an important substrate for energy metabolism, glucose is correlated with blood sugar levels in sweat, which can reflect the body’s energy supply and demand [50]. Cortisol is one of the core hormones that regulate nutrient metabolism and directly affects the body’s nutrient intake [51]. Moreover, glucose regulation combined with the effect of cortisol on appetite is a crucial factor in obesity [52]. Therefore, wearable sensors can jointly detect the concentration of glucose and cortisol in sweat, which can achieve a multi-dimensional assessment of human energy metabolism and assist in lifestyle management [53].

Another important multi-modal sensing strategy is to simultaneously detect the pH and metabolite concentrations of sweat. Fluctuations in sweat pH can directly affect the response characteristics of some electrochemical sensors to metabolites, resulting in drift in the measurement signal. For example, enzyme-based metabolite detection methods are significantly affected by pH, which greatly affects enzyme activity [40]. Xu, Z. et al. developed a sweat sensing patch that simultaneously detects pH and tyrosine concentration, and verifies the detection results of tyrosine through pH value, improving the accuracy of the sensor [36].

At present, there are two main ideas for the research of sweat multi-marker sensing. Some studies focus on demonstrating the versatility and integration capabilities of sensor devices, and have a wide range of coverage in marker selection. The other part of the study focuses more on application scenarios and explores the synergistic value of specific marker combinations in solving specific physiological or clinical problems. Nevertheless, a notable shortcoming in the current landscape is that some studies lack a clear physiological or clinical rationale for their chosen marker panels, occasionally combining multiple sensing modalities primarily to showcase platform generality. This strategy, which prioritizes demonstrating technological capability, often fails to fully elucidate the intrinsic correlations and synergistic diagnostic value among the selected markers, thereby limiting the depth and translational potential of the findings to some extent. Future work should place greater emphasis on a “needs-driven” design principle, promoting the integration of high-performance sensing platforms with marker panels designed based on clear physiological or clinical logic. This will enable sweat-based multi-modal sensing technology to serve key application scenarios, such as personalized health monitoring and disease-risk warning, in a more precise and effective manner.

#### 2.1.2. Other Sources of Body Fluid

In addition to sweat, other body fluids such as saliva, tears, and interstitial fluid are also rich in a variety of biomarkers and are important targets for multi-marker detection.

The levels of glucose, vitamins, nitrites, proteins, and other substances in tears can effectively reflect the health of the human body. In addition, tears directly infiltrate and protect the surface of the eyeball, and their biochemical composition can directly reflect the microenvironmental state of the ocular surface, so it can sensitively indicate the local health status of the eye. For example, nitric oxide is a key mediator for maintaining ocular homeostasis. As a metabolite of nitric oxide, the concentration of nitrite in tears is closely related to uveitis, retinitis, glaucoma, and other eye diseases [54]. The smart eye patch developed by Xu J. et al. can assist in the evaluation of human diabetes and eye lesions by using specific chromogenic reagents to achieve continuous monitoring of hydrogen ions (pH), protein, ascorbic acid, and glucose in tears [42].

Saliva contains important biomarkers such as glucose, lactic acid, nitrite, thiocyanate, and uric acid, which can be used for monitoring a variety of diseases. Moreover, saliva, as a body fluid that exists directly in the oral environment, can more effectively reflect the health of the oral cavity. For example, nitrite, as a stable metabolite of nitric oxide, has been shown to be associated with diseases such as dental caries, periodontal disease, and oral cancer [55,56]. Lucas F. de Castro et al. integrated a microfluidic paper device into a mouthguard to enable real-time monitoring of glucose and nitrite concentrations in saliva. By comparative analysis of saliva samples from healthy individuals, periodontitis or diabetic patients, the clinical application potential of the sensing system in the monitoring of oral and metabolic diseases is verified. Notably, this work exhibits good accessibility. By employing a low-cost, disposable paper-based microfluidic sensor integrated into a user-friendly mouthguard, the system requires no external equipment or specialized operation [43]. This approach highlights a viable pathway toward affordable and easy-to-use wearable devices, which is crucial for expanding health monitoring to broader populations.

ISF acts as a body fluid that is highly similar to blood composition [57], and it is an important source of clinical information. ISF can be collected through a microneedle array penetrating the skin, and then its pH, lactic acid, alcohol, glucose, and other indicators can be detected. For example, the simultaneous detection of glucose and lactate in ISF can aid in the management of related diseases and help advance our understanding of complex metabolic changes in the human body [45]. The combination of alcohol and glucose can assist in the treatment of alcohol-dependent patients and the detection of alcohol-induced health complications [44].

Currently, multi-analyte sensing technologies for a single biofluid are, on the whole, still in a transitional phase from laboratory prototypes toward validation in controlled environments. While numerous studies have preliminarily verified their feasibility in human trials, and some have begun to focus on clinically relevant metrics with preliminary designs for integrated clinical application [29], the vast majority of systems remain at the preclinical research stage. To advance genuine clinical translation, future work must urgently overcome several core bottlenecks: First, enhancing the long-term stability and anti-interference capability of sensors in real-world usage scenarios. Second, shifting the research paradigm from “technical validation” to “clinical validation” by designing rigorous clinical trials to establish quantitative correlations between multi-dimensional sensing data and specific health or disease states, ultimately realizing their practical value as tools for assisted diagnosis or management.

### 2.2. Coupling Acquisition of Body Fluid and Other Physiological Signals

In wearable multi-modal sensing systems, the simultaneous collection of humoral biochemical information with other physiological signals can provide a more comprehensive perspective for understanding the complex state of the human body. At present, research cases have emerged that combine bioelectrical signals, human movement information, skin temperature, and cardiovascular parameters with body fluid sensing (Table 2).

#### 2.2.1. Bioelectrical Signals

Capturing the synergistic changes and internal correlations between bioelectrical signals and body fluid information can overcome the limitations of a single signal source and provide more comprehensive and reliable physiological insights for health monitoring.

The synchronous monitoring of electromyogram (EMG) signals and sweat metabolites can reflect the muscle activity of the human body from the two complementary dimensions of neural activity and energy metabolism, which is of great significance in the field of sports science management and rehabilitation medicine. EMG signals encode information about the electrical activity of active motor units in the detection area [67], while the concentration of metabolites in sweat can indirectly reflect the energy supply and metabolic status during muscle activity [68,69]. In this direction, Shi, S. et al. developed an intelligent bionic skin patch. The device integrates the sensing of sweat metabolites with the detection of skin temperature, skin impedance, and EMG signals, so as to realize the comprehensive monitoring of the body’s energy metabolism and muscle activity during exercise. The single-guide sweat nanofiber membrane in the patch design can not only efficiently collect sweat samples for sweat metabolite sensors, but also effectively reduce the interference of sweat accumulation on surface EMG signal acquisition, and improve the quality of EMG signals. This multi-modal sensing strategy provides a powerful technical tool for sports performance assessment and rehabilitation process monitoring. The work also notes that such a multi-modal detection strategy advances the development of human health management [66]. Unlike traditional, discrete clinical checkups that require individual medical examinations, wearable sensors offer a far more convenient approach by enabling continuous, real-time, and multi-parameter monitoring, thus providing a practical technological foundation for large-scale, predictive health screening.

Electrodermal activity (EDA), or galvanic skin response (GSR), refers to the change in skin conductivity when the body is stimulated, and is often used to measure a person’s emotional response [70]. Emotional reactions can also trigger complex reactions in the endocrine system, affecting glucose, lactic acid, and synthesis of metabolites such as uric acid, affecting sweat composition [71]. Therefore, multi-modal sensing combined with the two can effectively monitor people’s psychological state. Xu, C. et al. quantified a high level of confidence in psychological stress responses through non-invasive monitoring of human sweat and three vital signs (pulse, EDA, and skin temperature). Among them, six biomarkers (glucose, lactic acid, uric acid, sodium ions, potassium ions, and ammonium ions) that are strongly related to stress response were selected as targets for sweat sensing [65].

Electrocardiogram (ECG) is a very important means of predicting and diagnosing cardiovascular diseases [72]. The epidermal biosensing patch developed by Md Abu Zahed et al. realizes the joint monitoring of electrocardiogram and sweat pH, temperature, and glucose concentration through the integration of glucose sensors and biopotential electrodes [60].

#### 2.2.2. Human Movement Information

In wearable multi-modal biochemical sensing systems, the integration of human motion signals greatly expands the application dimension of biochemical sensors. For example, simultaneous monitoring of human movement and sweat information is of great significance for comprehensively assessing health status and optimizing sports training strategies [73]. By monitoring the strain signals generated by limb movements, the system is able to distinguish between different types of physical activity. The concentration of sweat markers collected simultaneously reflected the changes in energy metabolism during exercise. Zhao, T. et al. developed a wearable biosensor based on retractable fibers, which integrates real-time sweat composition analysis and body motion capture, and cooperates with triboelectric nanogenerators (TENG) to build a self-powered closed-loop health monitoring system [61].

This multi-modal sensing strategy also shows rich possibilities for expansion. The wearable hydrogel sensor developed by Wei, J. et al. can accurately capture the subtle deformation of human activities on the basis of temperature sensing and sweat secretion monitoring. Based on this, the sensor can recognize handwritten numbers and letters, and can detect subtle human movements such as swallowing, speaking, and facial smiles, demonstrating the great potential of wearable multi-modal biochemical sensing systems in the field of electronic skin applications [64].

#### 2.2.3. Skin Temperature

Skin temperature and sweat secretion are closely related to the body’s heat regulation and are closely linked through shared physiological mechanisms. Sweating is the main mechanism by which the body dissipates heat and regulates body temperature while skin temperature is a direct reflection of the body’s heat status and also a key factor driving the sweat response [74]. Therefore, it is necessary to monitor skin temperature simultaneously with sweat kinetic parameters (e.g., sweat output, sweat flow rate, etc.), which can realize a comprehensive body assessment and identify health conditions such as heat stress, dehydration, and thermoregulation dysfunction. The flexible wireless sensing platform developed by John A. Rogers’ team combines thermistors with flow rate sensors to detect sweat flow rate, cumulative loss, and temperature, which can provide key data for identifying thermoregulation disorders and heat stress-related diseases.

In addition, at a technical level, the data of skin temperature and sweat secretion form a bidirectional calibration. Temperature is a key parameter to correct sweat biochemical sensor readings [58], and kinetic indicators such as sweat flow rate also help to interpret skin temperature data more accurately [75]. The multi-modal measurement of both can effectively improve the accuracy and reliability.

#### 2.2.4. Cardiovascular Parameters

Cardiovascular parameters, including heart rate, pulse, and blood pressure, are indicators of human vital signs. Fluctuations in fluid composition can directly or indirectly regulate blood pressure and heart rate by affecting mechanisms such as biosignaling molecules [76,77]. Moreover, by capturing the macroscopic parameters of the cardiovascular system and the microscopic biochemical information of body fluids in parallel can reveal human daily activities or disease states. A non-invasive wearable device developed by Juliane R. Sempionatto et al. enables real-time monitoring of blood pressure, heart rate, and multiple biomarkers (e.g., glucose, lactate, caffeine, alcohol) in body fluids by integrating ultrasound transducers and biochemical sensors. With this device, it was possible to successfully capture the synergistic effect of alcohol and glucose intake on increased blood pressure and heart rate due to hypoxia and lactate production during exercise. Specifically, blood pressure and heart rate are monitored via ultrasonic transducers, while the biochemical analytes are detected by electrochemical sensors. These distinct sensing modalities are integrated onto a single, flexible platform that is mechanically optimized for resiliency and conformity to curved skin surfaces, ensuring reliable operation without crosstalk between the sensor units [78].

### 2.3. Multi-Source Body Fluid Collection

The multi-source body fluid collection strategy aims to build a more comprehensive individual health assessment network (Table 3). Huang, M. et al. developed an ultra-wide linear range wireless ammonium ion sensing patch that can universally detect ammonium ion concentrations in tears, saliva, sweat, urine, and blood. Studies have verified that the patch has the ability of in situ wireless monitoring of human sweat and has been successfully applied to the analysis of blood ammonium in mouse models. For the remaining body fluids, the sensor has also passed the ex vivo sample test, demonstrating its broad applicability for cross-media detection [79].

Although some multi-source sensing systems have been developed, similarly to Huang et al.’s system, tears, saliva, etc., still need to be sampled in advance, highlighting the core difficulty of in situ sampling. Here, microfluidic chips represent a pivotal technology for in-situ body fluid sampling, enabling the automated, on-demand capture and transport of minute fluid volumes directly at the source (e.g., from sweat glands or the ocular surface). Recent advancements in flexible and wearable microfluidics have progressed toward systems capable of handling multiple analytes or even interfacing with the body for continuous sampling. At present, a team has developed a device that can be attached to the eye or placed in the mouth to perform in-situ sensing of a single fluid, and how to integrate these mature single fluid sensing modules into a unified system will be an important direction for the development of multi-source body fluid sensors. A promising technical approach is the “multi-module collaborative sampling + microfluidic channel interconnection” paradigm. This paradigm envisions a wearable platform where spatially distributed, self-contained sensing modules—each performing localized sampling and preliminary analysis of tears, saliva, or sweat via integrated microchannels—wirelessly transmit their data to a central processing unit or hub. There, advanced algorithms would integrate and analyze the multimodal data streams, uncovering cross-body correlations and generating a comprehensive health assessment. Moreover, this pursuit underscores the need for advanced modular design in sensor platforms—a challenge and its potential solutions will be elaborated in Section 3.2.2 on platform integration.

**Table 3:** Summary of multi-source body fluid sensor: devices, body fluids, and analytes and performance parameters.

Year	Device/Study	Body Fluid	Analyte	Performance Parameters
2021	Epidermal patch for the simultaneous monitoring of hemodynamic and metabolic biomarkers [78]	Sweat	Lactate	Feasibility validation during exercise
			Caffeine	Feasibility validation during caffeine intake
			Alcohol	Feasibility validation during alcohol consumption
		Interstitial fluid	Glucose	Feasibility validation during food intake
2022	A machine learning-based multi-modal electrochemical analytical device based on eMoSx-LIG [80]	Sweat, saliva	Tyrosine	Limit of detection: 116 μM (SWV), 21 μM (DPV) Sensitivity: 4.1 mAM^−1^ (SWV), 4.5 mAM^−1^ (DPV)
			Uric acid	Limit of detection: 3.5 μM (SWV), 1.2 μM (DPV) Sensitivity: 27.8 mAM^−1^ (SWV), 19.6 mAM^−1^ (DPV)
2022	Ammonium sensing patch with ultra-wide linear range and eliminated interference [79]	Sweat, tear, saliva, urine, blood	NH^4+^	Linear range: 10 μM–100 mM (sensitivity: 58.7 mV/dec)
			K^+^	Linear range: 1–20 mM (sensitivity: 61.5 mV/dec)
2023	Single fully integrated wearable sensor arrays for wirelessly, noninvasively, and simultaneously measuring [81]	Sweat, saliva, urine	Uricacid	Linear range: 0.005–0.6 mM and 0.6–4.5 mM Limit of detection: 1.42 μM
			pH	Linear range: 3–8 (sensitivity: −59.65 mV/pH)
			Na^+^	Linear range: 5–320 mM (sensitivity: 58.73 mV/dec)

## 3. Platform-Integration Mechanism

Different sensors enable the monitoring function of the multi-modal body fluid systems. Therefore, the response and sensing mechanisms of various types of sensors play a pivotal role in functional implementation, determining the accuracy, sensitivity, and stability of physiological signal acquisition. Current mainstream sensing mechanisms primarily fall into three categories: biochemical sensing, physical sensing, and biochemical-physical coupling mechanisms. As the functional implementation carrier, sensors’ practical applications rely on system integration. Based on integration level, these mechanisms can be classified into two types: monolithic integrated platforms and modular combination platforms. The former integrates sensors, signal processing, power management, and wireless communication components onto a substrate, emphasizing integration and miniaturization. Flexible substrates—known for their stretchability and biocompatibility—are currently the mainstream choice for daily skin monitoring applications. The latter adopts a “plug-and-play” approach by modularizing sensing units, offering enhanced scalability, flexibility, and ease of maintenance. This section focuses on the working principles, characteristics, and applications of sensing mechanisms, along with typical examples of both architectures.

### 3.1. Sensing Mechanism

#### 3.1.1. Biochemical Sensing Mechanism

Biochemical sensing mechanisms operate through the specific binding of biomolecular identifiers to target analytes, converting analyte indicators into measurable signals. A common design involves immobilizing highly specific biomolecular identifiers (such as enzymes, aptamers, antibodies, or artificial receptors) on the sensor surface. When target molecules bind or react with these identifiers, they trigger electrochemical, resistive, or optical signal changes. Signal acquisition and algorithmic processing are achieved through current measurement, optical inspection, or reference voltage calibration to determine analyte concentrations. This mechanism offers high sensitivity and selectivity. Currently, the primary biochemical sensing approaches are enzyme-catalyzed reactions and molecular binding mechanisms [40,82,83,84].

The enzyme-catalyzed reaction mechanism utilizes specific enzyme sensors targeting different analytes. Through enzymatic catalysis, the concentration signal of the target substance is converted into an electrical signal. For instance, glucose oxidase (GOx) catalyzes the oxidation of glucose to produce hydrogen peroxide, which undergoes electrochemical oxidation on the Pt working electrode surface. The generated current exhibits a linear proportionality with glucose concentration, enabling in situ, real-time, and quantitative detection of glucose in sweat [85]. This mechanism offers continuous detection advantages, as enzyme reaction products can be rapidly released and regenerated, facilitating continuous catalytic cycles. Such enzyme-based electrochemical biosensors typically employ a three-electrode system to record redox reaction currents under constant potential, thereby calculating analyte concentrations [86]. Currently, enzyme-based wearable sensors have achieved simultaneous detection of multiple metabolites in sweat. Gao et al. developed a flexible patch integrating enzyme electrode arrays for glucose and lactic acid detection, supplemented with skin temperature sensing for error correction, enabling simultaneous detection of multiple components in bodily fluids [85]. The patch combines flexible circuitry for signal processing and wireless transmission, allowing real-time assessment of the wearer’s metabolic status during physical activity. In order to improve the reliability of continuous detection of enzymatic sensing in vivo, Yang et al. reported a wearable differential microneedle array patch: Prussian blue/glucose oxidase was modified layer by layer on the surface of 160 μm stainless steel microneedles to form a three-electrode system. The microneedle patch is only 11 g, which can be inserted 1 mm into the epidermis without pain to obtain tissue fluid, so as to realize continuous blood glucose monitoring at 3 min intervals [87]. Due to their high signal conversion efficiency and sustainability, enzyme-catalyzed reactions have become the mainstream solution for bioanalytical sensing in bodily fluids. However, enzyme activity remains susceptible to environmental factors like temperature and pH, and their limited lifespan restricts long-term stability. To improve the environmental adaptability and reusability of enzymes, researchers generally adopt immobilization strategies, such as enzyme entrapment, covalent attachment, or nanoparticle support, which effectively enhance the structural stability of enzymes and reduce the loss of activity [88].

**Figure 2:**
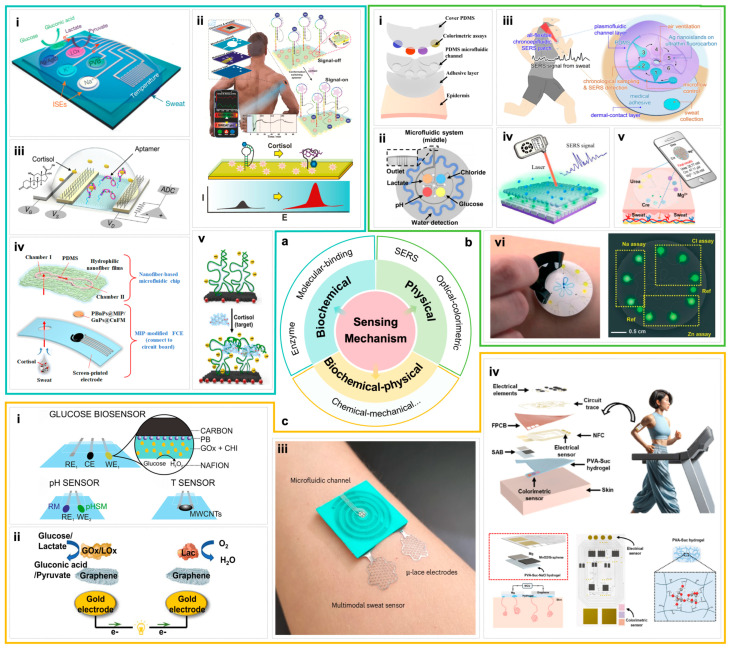
(**a**) Body fluid sensor based on biochemical sensing mechanism. (**i**) A flexible patch integrating an array of enzyme-based electrodes, which enables spatially distributed detection of ions, glucose, and lactate in different regions, supplemented by skin temperature sensing for error correction [85]. (**ii**) A cortisol DNA aptamer structure, through the target-induced pseudoknot-assisted conformation-switching aptamer, to achieve cortisol concentration detection. The cortisol concentration and sweat pH measurements can be transferred wirelessly for display and analysis on a nearby smart device [89]. (**iii**) A flexible field-effect transistor array fixed with a cortisol aptamer modulates the transistor current when cortisol in sweat is combined with it [90]. (**iv**) A flexible wearable electrochemical sweat cortisol sensor was constructed by nanofiber microfluidic chip and gold nanoparticle-doped MIP film [91]. (**v**) Cortisol binds to the aptamer and undergoes conformational changes [94]. (**b**) Physical sensing mechanism and examples of body fluid sensing. (**i**) PDMS microfluidic sensor patch structure equipped with optical colorimetric arrays [95]. (**ii**) A soft, wearable microfluidic sweat monitoring device that immobilizes colorimetric reagents for biomarkers such as pH, chloride ions, lactic acid, and glucose. When the sweat enters the channel, a specific reaction occurs with the reagent, causing a color change [96]. (**iii**) All-flexible CEP-SERS patch: plasmonic nanoislands on PDMS collect and time-sequence sweat via capillary micro-valves for label-free skin-conform metabolite profiling [97]. (**iv**) Schematic illustration of in situ detection of sweat based on SERS [98]. (**v**) The colorimetric results of in situ detection of sweat were analyzed by mobile phone application [99]. (**vi**) By comparing the fluorescence intensity, the concentration of the analyte was detected [24]. (**c**) Biochemical-physical coupling sensing mechanism and application. (**i**) Working mechanism of a skin patch based on the pH value and temperature correction of local sweat dynamic fluctuations in the body test is used for blood glucose analysis of sweat. Enzyme activity is greatly dependent on pH and temperature, and this correction significantly improves data reliability [58]. (**ii**) Working principle of a biosensor based on biofuel cell [86]. (**iii**) Sweat secretion rate sensor and μ-lace electrode based on microfluidics show a strong correlation between skin and forearm sweat secretion rate [100]. (**iv**) Multi-modal sweat monitoring equipment, which realizes self-power supply through sweat-activated batteries, can display in real time and perform long-term data analysis. The device integrates advanced colorimetric and electrochemical sensor arrays to measure pH, glucose concentration, and chloride ion levels in sweat, store these data and transmit them wirelessly through NFC [101].

Molecular binding mechanisms rely on specific interactions with target molecules, including nucleic acid aptamers and molecularly imprinted polymers (MIPs). Aptamer sensing utilizes artificially designed short nucleic acid chains (DNA or RNA oligonucleotides) that fold into specific structures to selectively bind target molecules with high affinity. This binding process induces structural or charge distribution changes in the aptamer, which can be detected and converted into signals through electrochemical or optical methods. Singh, N. K. et al. employed an artificially designed cortisol DNA aptamer that achieves cortisol concentration detection through target-induced pseudoknot-assisted conformation-switching aptamer (Figure 2a). In its free state, the aptamer forms a rigid pseudoknot structure with the molecular switch (MB) away from the gold electrode surface, resulting in extremely low background current. When cortisol in sweat binds to the aptamer, the pseudoknot is pulled apart, causing the aptamer to fold into a single stem-loop configuration. This brings the MB closer to the electrode, significantly enhancing electron transfer rates and generating a current response proportional to cortisol concentration [89]. Wang, B. et al. developed a flexible field-effect transistor array immobilized with cortisol aptamers (Figure 2a). By modulating transistor current upon cortisol binding in sweat, they achieved nanomolar-level ultra-low concentration detection and integrated the system into a wristwatch for real-time continuous monitoring of stress hormone levels [90]. MIP sensing involves pre-introducing template molecules into a polymer matrix, which are then polymerized and removed to create selective binding “imprint” cavities for target molecules. When the target molecule enters the cavity and binds to it, the electrochemical or optical signals at the interface undergo changes that can be used for quantitative analysis. Mei, X. et al. developed a wearable electrochemical sweat cortisol sensor by forming a gold nanoparticle-doped MIP film through electropolymerization on flexible electrodes (Figure 2a). This achieved a wide detection range from picomolar to micromolar levels with high selectivity, and demonstrated minimal signal attenuation during prolonged use [91]. The aptamer-MIP mechanism expands detection pathways for analytes such as small molecules and biotoxic substances that are challenging to detect using enzyme sensors. Compared to natural enzymes and antibodies, these two approaches exhibit superior environmental stability, longer shelf life, and lower costs, making them ideal candidates for wearable sensing applications [92]. Aptamer sensors demonstrate high stability and can be designed for multiple targets, but require consideration of non-specific interference when applied in complex matrices like body fluids. Molecularly imprinted polymers (MIPs) offer low-cost and robust materials, though their affinity and recognition efficiency at imprint sites still require further optimization [93]. Molecular binding mechanisms provide diverse options for biochem sensing in body fluids, achieving enhanced detection performance, improved stability, and reduced costs compared to enzymatic mechanisms.

#### 3.1.2. Physical Sensing Mechanism

Unlike biochemical sensing mechanisms, physical comparison sensing relies on the physical properties of target molecules to determine their concentration through measured signals. This approach requires no direct electrochemical interaction with the target molecules, features a relatively simple design, and can be integrated with portable detection devices. Current mainstream methods include optical techniques (such as color changes and fluorescence intensity variations) and Raman spectroscopy enhancement technique.

Optical colorimetric sensing primarily measures target molecule concentrations by analyzing color or light intensity changes. This method typically employs chemical indicators, such as pH indicators or enzyme reactions producing reactive dyes, that undergo specific reactions with target molecules, causing color variations. Users can visually observe or photograph these color changes to estimate concentrations. Koh et al. developed a soft, wearable microfluidic sweat monitoring device featuring colorimetric reagents immobilized in microfluidic channels and reservoirs for detecting biomarkers like pH, chloride ions, lactic acid, and glucose (Figure 2b). When sweat enters the channels, it specifically reacts with these reagents, triggering color changes. Users can then capture device images via smartphone cameras and analyze color intensity using integrated image processing to quantitatively assess target substance concentrations [96]. The optical colorimetric mechanism offers intuitive, cost-effective advantages for multi-parameter parallel detection and single-use applications. However, its limited precision and susceptibility to environmental light and skin color interference necessitate critical improvements in device encapsulation, sensitivity calibration, and signal correction. An example is the sweat sensor patch developed by Bandodkar, A.J et al., which combines colorimetric methods with digital image analysis to eliminate visual estimation errors. By capturing smartphone images and calibrating color references, the device converts color changes into precise concentration values. Furthermore, the study systematically evaluated the impact of different environmental lighting conditions. By introducing reference color charts and implementing image standardization, the stability and accuracy of colorimetric results were significantly enhanced. For instance, in chloride ion detection, the luminance parameter from the Lab color space was adopted for quantitative analysis, achieving linear response to chloride ion concentrations within the 20–160 mM range with maximum relative standard deviation controlled below 2.6% [102]. These findings demonstrate that while colorimetric methods are inherently susceptible to interference from ambient light and skin pigmentation, practical improvements in measurement accuracy can be effectively achieved through optimized encapsulation, color calibration, and image processing algorithms.

The Surface-Enhanced Raman Scattering (SERS) mechanism leverages localized surface plasmon resonance (LSPR) generated by noble metal nanostructures under laser excitation, significantly amplifying Raman scattering signals of target molecules. This enables “fingerprint” detection of trace analytes, allowing clear identification and quantitative analysis of characteristic Raman peaks. Wang et al. developed a flexible wearable SERS sensor based on sulfated cellulose-silver nanoparticle composite hydrogel for detecting urea, uric acid, and pH in sweat. The hydrogel’s porous structure efficiently adsorbs sweat, while 4-MBA functionalization enables pH-responsive detection across the typical adult sweat pH range (5.5–7.0), matching commercial pH meter results. The substrate maintains SERS activity for 30 days, demonstrating excellent stability and biocompatibility for long-term skin application [103]. Pan et al. further integrated three-dimensional chiral plasmonic structures into a wearable SERS platform, creating a microfluidic patch for real-time in situ monitoring of chiral metabolites in sweat (Figure 2b). Their 3D Ag nanorods/nanocubes bilayer structure forms high-density “hot spots” through interlayer coupling, enabling enantioselective detection of levofloxacin with a detection limit as low as 0.038 μM, highly consistent with HPLC results. The device also integrates pH sensing functionality, enabling real-time monitoring of drug metabolism dynamics during motion, demonstrating the immense potential of Surface-Enhanced Raman Energy Spectroscopy (SERS) in personalized drug monitoring and disease management [98]. A single SERS substrate can simultaneously detect multiple physical Raman spectra, enabling high-accuracy multi-parameter analysis. However, SERS wearable sensors still face challenges such as substrate preparation consistency, signal stability, and interference from complex bodily fluid backgrounds. Current improvement directions include developing flexible, stretchable, and mass-producible nano-substrates, introducing internal standard calibration, and combining microfluidic pretreatment to reduce noise [104].

In addition, there are pure electrochemical sensors, such as impedance-type and potential-type, which do not require biological recognition elements and directly output signals through interface capacitance or ion-selective membrane potential changes. The structure is simple and the stability is high. It has been used for real-time monitoring of sodium and potassium ions in flexible sweat, and can act as a non-enzymatic, long-life supplement module [105].

#### 3.1.3. Biochemical-Physical Coupling Mechanism

The biochemical-physical coupling mechanism leverages biochemical and physical sensing principles to harness synergistic advantages of multi-modal sensors, acquiring more comprehensive and accurate body fluid data. Physical parameters (such as temperature, pH, and flow rate) significantly influence biochemical sensor outputs, and coupling physical sensing helps correct signal errors. Integrating different types of sensor signals enhances reliability and expands monitoring dimensions. In Bandodkar et al.’s study, real-time measurement of sweat flow rate and total sweat volume combined with enzyme sensor readings successfully improved the accuracy of glucose and lactate concentration assessment in sweat, achieving non-invasive monitoring closer to physiological blood levels [102]. Recent advancements in machine learning algorithms for multi-sensor data fusion have enabled dynamic correction of chemical sensor readings based on parameters like temperature, sweating rate, and exercise status, improving accuracy in complex scenarios [106]. Meanwhile, emerging biochemical-physical coupling strategies expand new approaches and functionalities in body fluid sensing. Chemical-mechanical signal coupling utilizes material deformation or mechanical changes induced by physicochemical reactions as sensing outputs [107]. Self-powered sensing coupled with closed-loop feedback also fully utilizes chemical energy in body fluid to achieve simultaneous monitoring and intervention. For instance, Huang et al. developed a biofuel cell powered by sweat lactic acid to serve as a sensor (Figure 2c), directly reflecting metabolic intensity [86]. These emerging coupling approaches hold promise for enhancing the autonomy and intelligence of sensing systems. However, most innovative concepts remain in experimental verification. Ensuring long-term sensing reliability and device stability under complex physiological conditions remains a critical challenge to overcome.

#### 3.1.4. Summary and Prospects

In summary, biochemical sensing, physical sensing, and biochemical-physical coupling mechanisms each possess distinct advantages and limitations in the construction of body fluid monitoring systems. Biochemical sensing mechanisms, characterized by high specificity and sensitivity, particularly enzyme-catalyzed reactions [85] and molecular binding technologies [90], occupy a core position in the precise quantitative analysis of biomarkers. However, the susceptibility of enzyme activity to environmental factors like temperature and pH, along with limited lifespan, remains a primary bottleneck restricting long-term stable application. In contrast, physical sensing mechanisms, exemplified by the microfluidic colorimetric device developed by Koh et al. [96] and the SERS patch designed by Pan et al. [98] offer non-contact, intuitive, or “fingerprint-level” optical detection methods. While featuring relatively simple structures and ease of portable integration, they face practical challenges such as interference from ambient light and skin pigmentation backgrounds, as well as consistency in nanostructure substrate preparation. Biochemical-physical coupling mechanisms represent an advanced form of future development. As demonstrated by Bandodkar et al.’s use of physical parameters to correct chemical signal errors [102] and Huang et al.’s self-powered biofuel cell [86], this multi-modal fusion not only significantly enhances data accuracy in complex physiological environments through synergistic effects but also expands system intelligence and autonomy. Although currently most technologies in this category remain in the experimental verification stage, transitioning from conceptual innovation to reliable long-term practical application constitutes a critical direction for future research.

### 3.2. Platform-Integration Architecture

The integrated platform architecture is the physical carrier of the realization of multi-modal sensing functions. According to the structural design and integration degree, it can be divided into two types: monolithic integrated platform and modular combination platform.

#### 3.2.1. Monolithic Integrated Platform

The monolithic integrated platform directly collects body fluids from the target area by integrating multiple sensors and sources into a single unit. This architecture emphasizes high integration and systematic design, with implementations including flexible patches, bionic electronic skin, electronic fabrics, and smart contact lenses et al.

**Figure 3:**
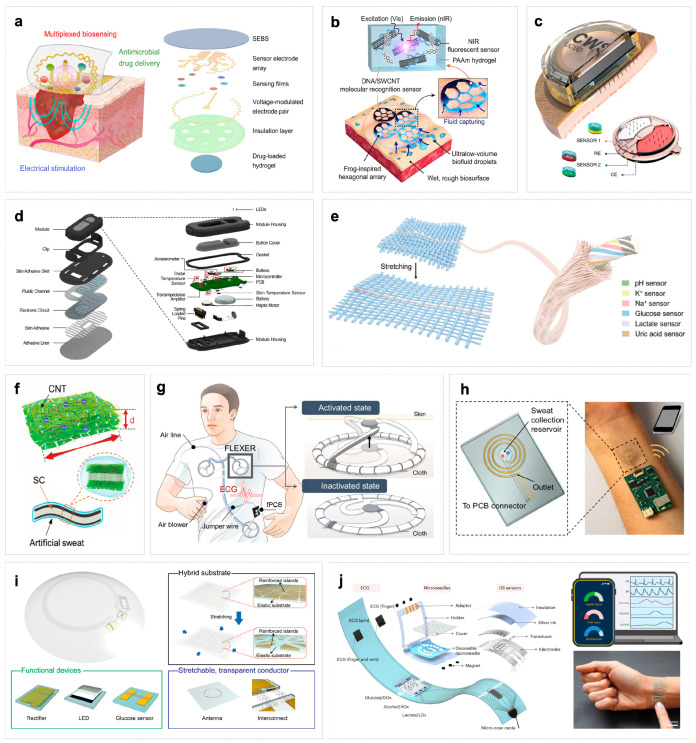
(**a**) Stable detection and wearable comfort are achieved by integrating the multilayer structure on a single substrate [108]. (**b**) Schematic illustration of the adhesion and molecular sensing detection mechanism of 3D MIN (3D microstructured patch integrated with optical nanosensor) on human skin [111]. (**c**) The multiplexed microneedles and sensors are integrated into the micro-patch structure [44]. (**d**) Exploded view of the device module and the underlying microfluidic substrate of a wearable microfluidic biosensor, including microfluidic channels, electrode circuits, and packaging [110]. (**e**) Flexible electronic fiber integrated with multiple sensors for multi-analyte detection in fabrics [40]. (**f**) Using MXene material and carbon nanotubes to make composite electronic skin can not only improve the stability of MXene, but also enhance the energy storage characteristics of electronic skin [112]. (**g**) A fabric-based layered MXene electrode for electrophysiological monitoring. By utilizing the high conductivity and low contact impedance of the MXene dry electrode, the electrode can provide stable skin–electrode contact, thereby achieving reliable biological signal detection in various situations [113]. (**h**) A wireless replaceable sweat detection patch. The spiral gold electrode is aligned with the microfluidic channel for sweat rate sensing, and the sodium sensor is located in the sweat collection tank [114]. (**i**) A flexible and intelligent contact lens that integrates a radio circuit, a glucose sensor and a micro display screen for real-time, non-invasive monitoring of glucose levels in tears [107]. (**j**) Explosive view with integrated BLUE platform, which integrates multiple sensors on its flexible substrate, can be used for simultaneous monitoring of multiple metabolites and cardiovascular signals in diabetic patients [115].

Flexible skin patches are among the most common forms, typically connecting sensing electrodes and signal processing circuits to an elastic substrate for conformal adhesion while enabling real-time fluid monitoring. For instance, Shirzaei Sani et al. developed a wireless wearable bioelectronic patch for chronic wound monitoring and treatment (Figure 3a). This device employs standard micro/nano fabrication techniques to create sensor arrays on a copper sacrificial layer, which are then transferred to a SEBS thermoplastic elastomer substrate, forming a highly flexible and stretchable patch structure. The patch integrates multiple electrochemical biosensors to monitor various biomarkers in wound exudate in real time, including glucose, lactic acid, uric acid, pH, temperature, and ammonium ions, while utilizing flexible printed circuit boards for signal processing and wireless communication [108].

Bionic electronic skin focuses on large-area coverage and multi-point physiological signal acquisition, utilizing bionic principles to achieve non-invasive fluid monitoring. Liu et al. systematically outlined the implementation pathways of the “lab-on-skin” concept, demonstrating how large-area flexible electronic skin can cover multiple skin regions. By integrating bionic structural designs, it enables real-time, non-invasive collection of physiological signals such as lactic acid, glucose, and ions in sweat [109]. These systems typically employ bionic microstructures or microfluidic channels to achieve in situ fluid collection and delivery (Figure 3d), while enabling multi-point, continuous monitoring [110]. Although numerous “electronic skin” sensing devices exist, most remain at the level of skin patch detection units without achieving large-area sensing. Multi-modal large-area electronic skin sensing still demonstrates promising application potential.

Electronic textiles integrate conductive fibers or printed circuits into fabric to achieve high breathability while maintaining comfort, enabling fluid sensing capabilities. For instance, Zhang et al. developed an integrated stretchable multifunctional electrochemical fiber combining helical carbon nanotube microelectrodes with superhydrophilic sheath layers (Figure 3e). This fiber not only exhibits excellent stretchability but also enables simultaneous detection of six biomarkers—including pH, K^+^, Na^+^, glucose, lactic acid, and uric acid—in trace sweat. When woven into breathable and moisture-permeable smart fabrics, it allows real-time continuous monitoring of human sweat composition [40]. Meanwhile, MXene materials have gained significant popularity in electronic textiles. Although most current studies have not directly achieved fluid component monitoring, existing work demonstrates their stable operation and responsiveness in fluid environments. Wang et al. proposed a “sweat-driven” electronic skin platform based on MXene-carbon nanotube composites (Figure 3f). This material directly utilizes sweat as an electrolyte while maintaining high conductivity and flexibility, achieving energy storage and thermal management functions. This achievement not only validates MXene’s excellent electrochemical stability and biocompatibility in sweat environments but also provides foundations for developing smart textiles with fluid sensing capabilities [112].

Furthermore, multi-modal fluid sensing systems often utilize daily objects as integrated platforms. For instance, Park et al. developed a flexible smart contact lens integrating glucose sensors, wireless circuits, and display pixels which can detect glucose concentration in tears in real-time and transmit results wirelessly, enabling non-invasive and continuous physiological monitoring [107].

#### 3.2.2. Modular Combination Platform

The modular combination platform allows functional modules to be combined in a decoupled manner to provide greater flexibility and scalability. Its core concept enables on-demand replacement of sensor components, enhancing system reusability and simplifying maintenance. A typical example is the split-type patch system (Figure 3h): disposable sensor patches handle initial detection through contact with bodily fluids, containing sampling interfaces and sensing elements (such as enzyme electrodes or ion-selective membranes), while reusable main control modules integrate batteries or energy harvesting units, signal conditioning circuits, and wireless communication systems connected via plug-and-play interfaces or magnetic contacts. This design allows only the inexpensive sensor patches to be replaced during each use, while the costly electronic modules remain durable, significantly reducing costs [114]. However, achieving on-demand expansion through plug-and-play or magnetic replacement interfaces to meet diverse application scenarios and individual needs still faces multiple bottlenecks.

#### 3.2.3. Architecture Comparison and Assessment

Regarding the selection of integration architectures, monolithic integrated platforms and modular combination platforms present distinct contrasts in cost-effectiveness, maintainability, and user experience. Monolithic integrated platforms, represented by the multifunctional wound monitoring patch by Shirzaei Sani et al. [108] and the electrochemical fiber by Zhang et al. [40], emphasize high structural integration and device flexibility. This architecture serves as an ideal carrier for high-compactness applications like “electronic skin” and smart contact lenses [107], offering superior wearing comfort, conformal contact, and seamless signal acquisition. However, high integration is often accompanied by higher manufacturing costs, and the monolithic nature limits device reusability since the entire unit must be replaced. Conversely, modular combination platforms (such as the split-type patch design [114]) decouple inexpensive disposable sensor interfaces from costly reusable signal processing circuits. This significantly reduces long-term user costs while endowing the system with flexible maintainability and functional scalability. Although modular designs still face bottlenecks regarding interface connection stability, standardization, and foreign body sensation control, their advantages in balancing high-performance monitoring requirements with economic feasibility grant them undeniable potential in large-scale population screening and personalized health management scenarios [21,116].

## 4. Data-Processing Pattern

The collected multi-modal data of different physiological signals need to be processed to be applicable. Compared with single modal data, multi-modal data allows for comparison, supplement, and fusion analysis. This section analyzes the multi-modal data-processing patterns from three different dimensions. First, cross-validation and calibration using different bodily fluid markers can significantly improve the accuracy and reliability of monitoring results [81]. Second, combining machine learning to analyze collected multi-modal information enables health prediction and personalization. Finally, data fusion integrates diverse information from different human systems, providing a more comprehensive physiological and pathological perspective, thereby enabling more precise and thorough tracking and evaluation.

### 4.1. Cross-Validation and Calibration of Multi-Modal Data

The traditional strategy of multi-modal body fluid monitoring is to carry out cross-modal correlation calibration and compensation based on independent physiological signals under the guidance of empirical formulas or parametric models [117]. Common multi-parameter cross-calibration methods are dynamic compensation based on state variables (such as temperature, pH, and sweat flow rate); cross-calibration between biomarkers and systemic indicators; and the role of structural and contact state monitoring in auxiliary calibration [117,118].

**Figure 4:**
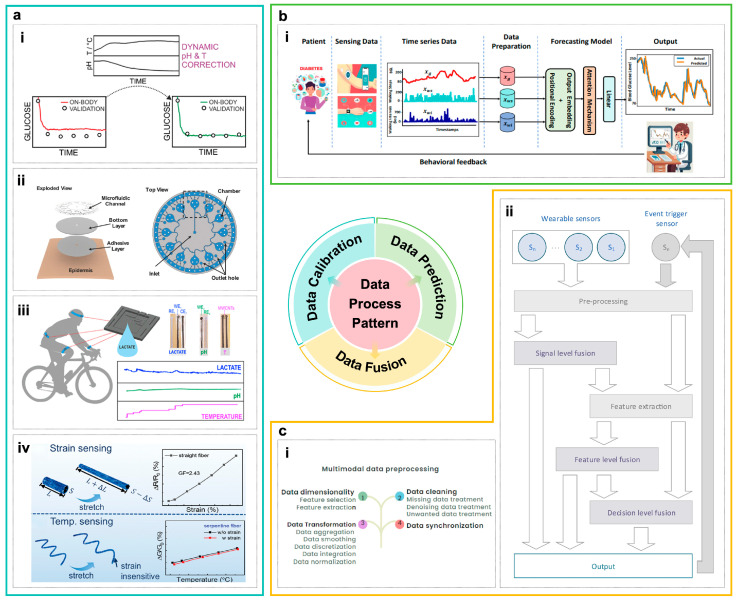
(**a**) Cross-validation and calibration of multi-modal data. (**i**) A T/pH–current calibration surface constructed for biomarker detection, utilizing real-time localized temperature and pH monitoring to effectively minimize errors during dynamic perspiration [58]. (**ii**) A microfluidic system to achieve on-chip separation of sequential sweat samples and a calibrated sweat volume–time mapping [120]. (**iii**) A novel flux-limiting strategy for lactate analysis, systematically validated through in vivo tests and Bland–Altman analysis under dynamic sweating, confirming its reliability for practical applications [121]. (**iv**) A design strategy for multi-module sensing, integrating structural mechanics and real-time status monitoring to mitigate interference and enable self-calibration [122]. (**b**) Machine learning and inference with multi-modal data. (**i**) An overview of the AttenGluco framework, illustrating its multi-branch Transformer architecture that integrates CGM signals with contextual data via cross- and multi-scale attention to achieve high-accuracy forecasting of glucose trajectories and hypoglycemia risks [123]. (**c**) Intelligent fusion and applications of multi-modal data. (**i**) An illustration of key multi-modal data preprocessing modules, demonstrating the procedures for data cleaning, synchronization, transformation, and dimensionality reduction [124]. (**ii**) A data fusion architecture for wearable health monitoring systems, which operates across three hierarchical levels: data, feature, and decision fusion [125].

Skin temperature and pH are commonly used as default calibration benchmarks in many enzyme/ion sensor array systems [85]. This mechanism relies on real-time compensation for dependence of enzyme activity and electrode potential on temperature and acidity. For enzymatic amperometric glucose sensors, temperature and pH directly influence enzyme activity and the diffusion coefficient. Wiorek et al. effectively mitigated errors arising during dynamic sweating by constructing a calibration surface that maps the combined influence of temperature and pH on the current output, with a point-by-point correction of the signal using real-time localized pH and temperature data (Figure 4a) [58]. For potentiometric electrolyte sensors, the influence of temperature on the Nernstian slope is well-defined. To address this, the Yeo team incorporated a skin temperature channel into their wireless flexible Na^+^/K^+^/pH array. This allows for the real-time calculation of temperature-compensated potentials based on the measured local temperature, thereby achieving stable and accurate monitoring of sweat electrolytes across a thermal stress range of 8–56 °C [119].

Sweat flow rate determines analyte concentration by altering dilution factors, residence time, and boundary layer thickness. This relationship allows the inferred instantaneous concentration to serve as an indicator of sweat “freshness”. Ursem et al. systematically delineated the critical role of impedance/capacitance-based flow rate sensors and evaporative or capillary-driven microfluidic structures in sweat analysis, stating that neglecting flow rate is mechanistically incomplete and can lead to misinterpretation of physiological status [126]. In terms of structural design, the integration of temporal blocks—such as CBVs, microfluidic channels, and chambers—enables the physical separation of sequentially secreted sweat fractions on-chip. This approach simultaneously establishes a well-defined mapping between the effective sampling volume and time (Figure 4a) [97,102,120].

Cross-calibration at the biomarker level often utilizes physiologically stable biomarkers or those with coupled transport mechanisms to correct for transient fluctuations in the target analyte. These sensor devices typically use multi-channel simultaneous measurements to monitor lactate, glucose, pH, and electrolytes. Koh et al.’s electrochemical energy–microfluidic skin patch has been capable of simultaneously measuring sweat lactate, glucose, Cl^−^, and pH, and compare these measurements with concurrent blood samples. It is already exploring a “composite indicator calibration” and risk characterization framework based on physiological mechanisms [96]. It is crucial to emphasize that the reliability and validation of sensor readings are paramount. Xuan et al. conducted a systematic validation of their lactate sensor. Furthermore, its reliability under dynamic sweating conditions was confirmed through repeated in vivo experiments and Bland–Altman analysis (Figure 4a) [121].

Furthermore, structural design and integrated monitoring of deformation, pressure, and contact status serve as an integral component for mitigating interference and supporting model-based calibration. On the one hand, the use of flexible, conformable materials [127,128] and electrode structures [129,130] helps maintain intrinsically stable electrical properties during bending, stretching, or localized slippage. On the other hand, improvements in the overall device architecture and morphology can create self-compensating architectures for parameters like multi-axial strain and temperature (Figure 4a) [122], thereby significantly suppressing mechanical artifacts and electrical noise arising from fluctuating contact impedance [131].

Most calibration approaches rely on semi-empirical formulas or parametric models grounded in physical mechanisms, such as temperature- or pH-based compensation. However, several studies are now incorporating machine learning and historical paired data analysis to learn more complex cross-calibration relationships. Sweat glucose signals, together with multi-channel vital signs, have been incorporated into a regression model for estimating blood glucose levels before and after exercise [132]. Similarly, existing work has early-fused lactate, sweat flow rate, heart rate, and individual exercise-related metadata within a multilayer perceptron (MLP) model to fit a nonlinear, multivariate sweat-to-blood lactate calibration surface [133]. This framework can be readily extended to other modalities.

### 4.2. Machine Learning and Prediction of Multi-Modal Data

Data-driven machine learning methods can facilitate intelligent inference and individual assessment in bodily fluid sensing systems by learning patterns and making predictions directly from data.

#### 4.2.1. Core Algorithmic Families and Their Complementary Roles

First, different algorithmic families play complementary roles in multi-modal bodily fluid and physiological monitoring. Supervised learning is most appropriate when reliable labels are available (e.g., disease diagnoses, arrhythmia classes, hypoglycemic events) and the objective is to learn a direct mapping from multi-modal inputs to these targets within a predefined prediction horizon [28]. By contrast, un-supervised and semi-supervised learning are better suited to scenarios where labels are scarce, noisy, or expensive to obtain—for example, when discovering latent physiological states, stress, or fatigue episodes, and inter-individual phenotypes from long-term wearable recordings [134]. In such cases, clustering and representation-learning methods can provide pseudo-labels or prior structure that subsequently guide supervised models. Neural network-based architectures (e.g., CNNs, RNNs, Transformers) are particularly advantageous for fusing high-dimensional, nonlinear, and long-range temporal dependencies across biochemical, cardiovascular, and motion signals [29], whereas linear or tree-based models remain competitive for low-dimensional feature spaces, near-threshold decision rules, or applications with stringent latency and energy constraints at the extreme edge [135].

Supervised learning methods are widely employed in multi-modal bodily fluid monitoring systems to train regression or classification models using labeled data. These models integrate multi-source information within sliding time windows to replace traditional single-sensor thresholds, thereby achieving more accurate predictions and reduced errors [136]. For instance, compared to relying solely on continuous glucose monitoring or simple threshold-based judgments, multi-modal machine learning achieves higher sensitivity and lower false positive rates in predicting events such as hypoglycemia [136]. Hong et al. modeled calibrated sweat glucose signals in combination with heart rate, blood oxygen saturation, and motion features, capturing fine-grained dynamic blood glucose fluctuations during exercise [132]. Mohapatra et al. developed a hierarchical fatigue prediction model based on multi-node inertial measurement units and vital signs, which more accurately captures subjective fatigue perceptions compared to simple threshold-based methods [137]. However, the performance of supervised learning methods is constrained by sample size, data quality, and processing pipelines. Therefore, cross-population generalization and large-scale deployment remain their primary challenges.

#### 4.2.2. End-to-End Pipelines and Performance Trade-Offs

In practical applications, the end-to-end data-processing pipeline typically encompasses the entire workflow, ranging from raw signal acquisition and multi-modal fusion to the final prediction of health status [25]. Taking glucose management based on the synergy between sweat and physiological signals as an example, a typical end-to-end processing pipeline can be summarized into the following steps:

Signal Acquisition and Synchronization: Synchronously collect sweat glucose signals, heart rate, blood oxygen saturation, skin temperature, and motion acceleration data [138,139].

Signal Preprocessing and Dynamic Calibration: Perform real-time compensation and correction on sweat glucose signals based on local temperature and pH values, as well as denoising and baseline correction on motion-related signals [140].

Feature Extraction and Cross-Modal Fusion: Extract temporal features of each modality using convolutional neural networks, and implement feature-level fusion via the cross-modal attention mechanism to capture the dynamic correlation between motion and metabolism [141].

Model Training and Trend Prediction: Adopt recurrent neural networks or Transformer architectures to model the fused features within sliding time windows, and output the blood glucose variation trend and hypoglycemia risk probability for the next 15–60 min [142].

Personalized Parameter Adaptation: Dynamically update model parameters based on user historical data with the aid of transfer learning or online learning strategies, so as to achieve the optimization of individualized prediction [143].

Such a pipeline not only enables the synergistic utilization of multi-source information, but also reduces system-level errors through end-to-end optimization, thereby enhancing the overall robustness in dynamic physiological environments [25].

In the context of end-to-end pipelines, the selection of algorithms inevitably involves performance trade-offs, which is particularly evident in ECG and broader wearable monitoring studies. Feature-based shallow models (e.g., linear regression, support vector machines, decision trees, random forests) typically require few parameters and incur low inference latency, making them attractive for implementation on microcontroller-class hardware or other low-power sensor nodes [30]. However, their performance often saturates when the input–output relationship is highly nonlinear or depends on extended temporal context, such as in the case of future cardiovascular risk or complex arrhythmia patterns [31]. Deep neural networks, including quantized and architecture-optimized variants, generally achieve higher accuracy and more robust event detection than threshold rules or shallow models, and can substantially reduce false positive rates in continuous monitoring settings [144]. These gains, however, come at the cost of increased multiply–accumulate operations, a larger memory footprint, and greater implementation complexity; thus, practical wearable deployment typically relies on model compression, quantization, and careful co-design with the target hardware platform [29]. Un-supervised and self-supervised representation-learning approaches occupy an intermediate position: although training is more involved, the resulting compact embeddings enable very lightweight downstream classifiers to preserve much of the predictive performance while supporting individualized calibration and population-level phenotyping in label-limited regimes [134].

#### 4.2.3. Advanced Models for Multi-Modal Fusion and Inference

Currently, multi-stream neural networks and temporal deep learning models are emerging as pivotal tools for multi-modal bodily fluid data fusion and intelligent inference, effectively suppressing noise and artifacts. A typical architecture involves separate feature extraction streams for each modality, followed by high-level fusion via cross-modal attention or gating mechanisms, which allows the network to adaptively weight features across modalities and time steps. For instance, in glucose management, Farahmand et al. proposed a Transformer-based multi-stream architecture that employs cross-attention and multi-scale temporal attention mechanisms to integrate CGM signals with contextual information such as physical activity and heart rate, achieving high-accuracy prediction of future 15 to 60 min glucose trajectories and hypoglycemia risk (Figure 4b) [123,145]. Similarly, Kasnesis et al. effectively fused PPG and accelerometer signals using cross-modal attention and modality-specific convolutions within a teacher–student framework, demonstrating robustness under intense motion interference. This approach provides valuable insights for the joint modeling of sweat lactate/electrolytes with metrics such as power output and step frequency [146].

#### 4.2.4. Key Challenges in Practical Applications

However, despite the strong representational capability of data-driven methods, they still face the challenges of overfitting and insufficient interpretability in practical applications [25]. In scenarios with small sample sizes or high noise levels, complex models (e.g., deep neural networks) tend to overfit the training data, leading to degraded performance on unseen data [147]. To mitigate this issue, regularization techniques (e.g., dropout, weight decay), data augmentation strategies (e.g., time warping, noise injection), and ensemble learning methods (e.g., model averaging, stacking generalization) can be adopted to improve the model’s generalization capability [147].

In addition, the decision-making process of black-box models (e.g., deep neural networks) often lacks transparency, which may affect clinical credibility and user acceptance in healthcare scenarios [148]. Notably, large-scale models, which often exhibit stronger predictive capabilities, tend to have even poorer interpretability, further exacerbating this issue. To enhance interpretability, the attention mechanism can be introduced to visualize the model’s key regions of interest for different modal signals [141], or explainable machine learning methods (e.g., SHAP, LIME) can be applied to conduct feature attribution analysis on prediction results [148]. Meanwhile, model compression and edge deployment technologies can migrate part of the inference process to local devices while maintaining performance, which not only ensures data privacy but also supports real-time feedback and interpretability output [149].

Beyond supervised learning, un-supervised and semi-supervised methods also play significant roles in processing multi-modal wearable data, particularly when labeled data are scarce and noise is prominent. These approaches can automatically identify underlying physiological states and individual differences. Typically, multi-channel physiological signals are first normalized and subjected to feature learning (e.g., using sequence autoencoders or self-supervised methods [150]). Subsequently, techniques such as Gaussian mixture models, spectral clustering [151], or fuzzy C-means [150] are applied in low-dimensional spaces to uncover underlying physiological states, such as stress episodes [152], emotional fluctuation intervals [153], or high-load physiological conditions [152]. These identified underlying physiological states and behavioral patterns can serve as training samples and prior information for subsequent supervised models [153] and support individualized calibration and population phenotyping [154]. However, the application of these methods in sweat biochemical sensing remains relatively limited. Maintaining clustering stability and result interpretability in data-sparse, high-noise environments with pronounced individual differences remains an unresolved critical challenge.

#### 4.2.5. Development Trends and Practical Training Loops

It is crucial to emphasize that data-driven approaches should evolve beyond single-instance offline modeling toward systems capable of supporting personalized modeling, online adaptation, and uncertainty quantification. Specifically, on the one hand, leveraging long-term historical data records, strategies such as transfer learning, incremental learning [155], or Bayesian regression can be used to dynamically update individual parameters. This enables the model to maintain population-level generalizability while achieving personalization. On the other hand, deep ensemble models or probabilistic forecasting networks can be utilized to explicitly output prediction intervals and confidence levels. These outputs can be integrated with sensor health status and signal quality assessments [156], ensuring that high-confidence decisions are output only when reliable signal integrity is maintained at the sensor frontend. With the maturation of edge computing and model compression techniques, such hybrid modeling frameworks—which integrate mechanistic priors with data-driven learning—are expected to perform most preprocessing and risk assessment locally on wearable devices [110], thereby achieving an optimal trade-off among energy consumption, bandwidth, privacy, and intelligence.

In real-world scenarios, training machine learning models must address challenges such as inconsistent data quality, limited samples, significant individual differences, and difficulty in obtaining annotations [16]. It is important to note that while the application of machine learning has been extensively discussed, these models must be trained on large-scale datasets to perform accurately. Researchers typically adopt targeted data collection strategies—for example, collecting high-quality time-series data through standardized motion protocols in controlled environments (e.g., Stetter et al. constructed a knee joint torque model using IMU data from 13 subjects) [157]. When labels are scarce, semi-supervised learning and multi-modal cross-validation (e.g., sweat-blood glucose pairing) can be leveraged to enhance supervision reliability. To improve model generalization, data augmentation (temporal warping, noise injection), transfer learning, and online personalized modeling are commonly used to adapt models to users’ dynamic physiological characteristics. Ultimately, models need to undergo long-term field testing and online calibration to verify their robustness, and integrate false positive suppression mechanisms such as that proposed by Rejab et al. to reduce false alarm rates and improve clinical usability. In summary, model training in real-world scenarios is a closed-loop process from data collection and algorithm optimization to continuous validation, relying on the close collaboration of sensor technology, clinical protocols, and learning algorithms [16].

### 4.3. Intelligent Fusion and Applications of Multi-Modal Data

Data fusion is an emerging paradigm for processing multi-modal sensor data streams. It moves beyond simple calibration or direct model input, enabling feature extraction from parallel, multi-dimensional, and heterogeneous data streams. This approach can provide a more comprehensive overview than what can be achieved with a single data source or with multiple, unintegrated sources [158,159]. For instance, quantifying glucose levels in sweat is useful while this offers limited value for assessing overall health or diagnosing diseases without the context of other sweat biomarkers, such as hormones, metabolites, and electrolytes. Furthermore, if multi-source data are merely presented in parallel without accounting for their correlations, it hinders the effective extraction of coherent latent states that represent overarching health trends.

Data fusion in multi-modal monitoring systems typically follows a systematic “Sensing-Preprocessing-Fusion-Inference-Feedback” framework [32]. Reference [124] provides a detailed overview of key preprocessing methods—such as dimensionality reduction, data transformation, synchronization, and cleaning—which constitute critical preparation before the fusion stage (Figure 4c). These steps are crucial for enhancing the quality of wearable data and, consequently, the overall effectiveness of data fusion.

The “fusion” stage is typically implemented at one of three levels: data-level, feature-level, and decision-level fusion (Figure 4c) [125]. Data-level fusion, which operates directly on raw sensor data, is simple to implement. Yet, it poses challenges for feature extraction when directly comparing disparate signals (e.g., from pH and glucose sensors). In contrast, feature-level fusion employs neural networks to extract shared multi-modal features, effectively leveraging complementary information across parameters. Nevertheless, it is prone to overfitting in small-sample scenarios and faces interpretability challenges [27]. Decision-level fusion combines the final predictive outputs from multiple modalities to inform a consolidated decision. This approach focuses on the ultimate inference but may overlook informative disparities in the underlying features [19].

In practical applications, the choice among data-, feature-, and decision-level fusion depends strongly on whether the modalities are homogeneous or heterogeneous. For homogeneous or same-source signals, such as multiple sensors targeting the same analyte (e.g., sweat glucose or lactate at different skin locations) or multi-channel measurements of a single physiological field (e.g., multi-lead ECG or combined ECG–PPG for heart-rate and arrhythmia monitoring), data- or feature-level fusion allows the system to exploit strong cross-correlations and shared noise characteristics and is therefore commonly implemented via multi-channel filtering, state-space models, or joint feature representations [160]. For example, in multi-modal ECG analysis, fusing time-, frequency-, and time–frequency-domain representations of the same ECG at the feature level has been shown to outperform late/decision-level fusion, highlighting the benefit of early integration when all inputs observe a common cardiac source [161]. By contrast, for heterogeneous signal combinations—such as integrating sweat ions, metabolites, and hormones with electrophysiological or hemodynamic signals (e.g., ECG, blood pressure, impedance cardiography)—the modalities differ substantially in physical units, sampling frequency, and noise models, so naïve data-level concatenation often leads to misalignment, non-stationarity, and high-dimensional, poorly conditioned feature spaces [162]. In such settings, multi-modal medical AI systems typically apply modality-specific preprocessing and modeling, and then combine outputs at the feature or, more conservatively, decision level (e.g., by averaging or weighting predicted risks), which better respects modality-specific error structures, is more robust to missing channels, and mirrors clinical practice in which ECG, laboratory tests, and imaging findings are first interpreted separately and only then integrated into a final diagnosis [163].

It is noteworthy that although data fusion represents a processing paradigm for multi-modal data distinct from cross-calibration and model-based prediction, its practical implementation does not entirely dispense with methods such as data calibration and machine learning. This is particularly relevant in the context of wearable medical devices for bodily fluid monitoring, where multi-modal data are highly susceptible to interference from environmental conditions, device crosstalk, and involuntary motion. The adoption of cross-calibration mechanisms to enhance data robustness is thus indispensable. Meanwhile, artificial intelligence analytical methods—including machine learning and deep learning—can actively contribute to the interpretation and fusion of information derived from multiple data sources [164]. These approaches are capable of discerning more complex data characteristics and supporting more accurate predictive, preventive, and personalized medicine (3PM) in monitoring, diagnosis, and therapeutic decision-making [165].

The value of multi-modal data fusion extends beyond simply adding data channels to the synergy achieved through multi-level processing, which enables effective noise suppression and physiological state reconstruction [19,32]. Moving forward, research must evolve beyond generic frameworks by integrating the dynamic properties of bodily fluids and individual differences. This will be crucial for developing robust, intelligent monitoring paradigms that offer reliable technological foundations for wearable health [27,32,117].

## 5. Conclusions

Wearable multi-modal body fluid monitoring systems provide a powerful foundation for non-invasive, continuous, and context-aware health monitoring. By integrating biochemical and physical information across multiple modalities, these systems overcome the limitations of single-parameter sensing and offer a more comprehensive and dynamic representation of physiological status (Table 4).

At the sensing level, combining multiple biomarkers within a single fluid, coupling biochemical signals with physiological parameters, and expanding towards multi-source fluid analysis collectively enhance the depth and reliability of health assessment. At the platform level, flexible patches, electronic skins, microneedle systems, smart textiles, and modular architectures have significantly advanced device integration, comfort, and scalability. At the data-processing level, cross-modal calibration, machine learning models, and multi-level data fusion substantially improve accuracy, resilience, and personalized interpretation. Emerging data-driven frameworks that incorporate historical records and uncertainty estimation further support individualized, adaptive, and trustworthy monitoring.

However, the development of multi-modal wearable body fluid sensing devices remains at an early stage of system-level research, and large-scale commercialization still faces numerous challenges. These include ensuring long-term biochemical stability, improving cross-user generalizability, enhancing large-scale manufacturability, reducing overall costs, and establishing unified multi-modal data fusion frameworks. Clinical-grade medical applications impose even more stringent requirements in terms of accuracy and response speed (Table 5), and they further demand sufficiently large clinical validation cohorts as well as more mature and comprehensive regulatory frameworks. Nonetheless, the convergence of advanced sensing mechanisms, integration technologies, and intelligent analytics is driving multi-modal wearable systems toward real-world, personalized, and predictive healthcare applications.

## Figures and Tables

**Table 1:** Summary of single-source multi-modal body fluid sensor: devices and analytes and performance parameters.

Body Fluid	Year	Device/Study	Analyte	Performance Parameters
Sweat	2019	Internally cross-validating multi-modal sweat sensing patch [35]	Na^+^	Linear range: 10–200 mM
			Sweat rate	Linear range: 0.5–3 μL/min
			Total ionic charge	Sensitivity: 0.063 μS mM^−1^
	2023	Wearable sweat sensor based on multifunctional conductive hydrogel [36]	Tyrosine	Linear range: 10–200 μM (R^2^ = 0.9985) Limit of detection: 3.3 μM
			pH	Linear range: 3.98–8.09 (R^2^ = 0.9975)
	2023	PPE(Photo-patternable Ecoflex) for intrinsically stretchable multi-biochemical sensor [37]	Glucose	Working range: 0.1–0.5 mM
			Lactate	Working range: 1–15 mM
			pH	Working range: 5–7
			Humidity	Qualitatively assessed
	2023	Dual-signal readout paper-based wearable biosensor [38]	Glucose	Linear range: ~10–250 μM (R^2^ = 0.997)
			Lactate	Linear range: ~2–25 mM (R^2^ = 0.991)
			Uric acid	Linear range: ~10–250 μM (R^2^ = 0.995)
			Magnesium ions	Linear range: ~0.5–5 mM (R^2^ = 0.992)
			Cortisol	Log-Linear Range: 0.1–10,000 mM (R^2^ = 0.994)
			pH	Linear range: 3–8 (R^2^ = 0.994)
	2023	Soft, environmentally degradable microfluidic devices for multi-purpose sweat sensor [39]	Sweat loss	Feasibility validation during exercise
			Sweat rate	Feasibility validation during exercise
			pH	Linear range: 4.5–7 (R^2^ = 0.985)
			Chloride	Linear range: 25–100 mM (R^2^ = 0.982)
	2024	Sweat phenylalanine multi-modal analytical biochips [9]	Phenylalanine	Working range: 10–1000 μM
			Chloride	Feasibility validation during exercise
			Sweat rate	Working range: 0.5–2 μL/min
	2024	All-in-one multifunctional and stretchable electrochemical fiber [40]	K^+^	Working range: 2–32 mM (sensitivity: 62.3 mV/dec) Limit of detection: 20.4 μM
			Na^+^	Working range: 10–160 mM (sensitivity: 49.1 mV/dec) Limit of detection: 79.1 μM
			Glucose	Sensitivity: 0.79 nA μM^−1^ Limit of detection: 4.2 μM
			Lactate	Sensitivity: 86 nA μM^−1^ Limit of detection: 0.4 mM
			Uric acid	Sensitivity: 3.4 nA μM^−1^
			pH	Working range: 4–7 (sensitivity: 56.8 mV/pH)
Tear	2019	Eyeglasses-based tear biosensing system [41]	Alcohol	Feasibility validation during alcohol consumption
			Vitamins	Feasibility validation during vitamin intake
			Glucose	Feasibility validation during food intake
	2022	Wearable Eye Patch Biosensor for Non-invasive and Simultaneous Detection of tears [42]	Protein	Limit of detection: 0.17 g/L
			Ascorbic acid	Limit of detection: 7.0 μM
			Glucose	Limit of detection: 3.0 μM
			pH	Measurement precision: 0.15 pH unit
Saliva	2019	Salivary diagnostics on paper microfluidic devices [43]	Glucose	Linear range: 0–2.0 mM (R^2^ ≥ 0.994)
			Nitrite	Linear range: 0–400 μM (R^2^ ≥ 0.994)
Interstitial fluid	2022	Integrated wearable microneedle array for the continuous monitoring of interstitial fluid [44]	Lactate	Dynamic ranges: 0–28 mM Limit of detection: 0.15 mM
			Glucose	Dynamic ranges: 0–40 mM Limit of detection: 0.32 mM
			Alcohol	Dynamic ranges: 0–100 mM Limit of detection: 0.50 mM
	2023	Wearable Sensor Patch with Hydrogel Microneedles [45]	Glucose	Linear range: 0.1–3 mM (sensitivity: 0.024 ± 0.002 μA mM^−1)^
			Lactate	Linear range: 0.1–12 mM (sensitivity: 0.0030 ± 0.0004 μA mM^−1)^
	2024	Laser-drilled hollow microneedle (HMN) patch for continuous sampling and sensing of interstitial fluid [46]	Glucose	Low conc. (0.02–1 mM): Sensitivity: 0.254 μA mM^−1^, Limit of detection: 5.6 μM High conc. (3–15 mM): Sensitivity: 7.1 nA mM^−1^, Limit of detection: 0.2 mM
			pH	Working range: 6.94–9.23 Linear range: 7.17–8.19 (sensitivity: 50.38 mV/pH)

**Table 2:** Summary of sweat–physiological signal sensor: devices, sweat analytes/physiological signals, and performance parameters.

Year	Device/Study	Sweat Analyte/Physiological Signal	Performance Parameters
2020	Dynamically calibrated epidermal patch for sweat glucose analysis [58]	Glucose	Linear range: 10–200 μM
		pH	Linear range: 4.5–8.1
		Temperature	Linear range: 19–43 °C
2021	Soft on-skin platform for wireless monitoring of sweat [59]	Sweat rate	Working range: 0–5 μL/min
		Sweat loss	Feasibility validation during exercise
		pH	Working range: 4.5–6.5
		Chloride	Working range: 12.5–100 mM
		Creatinine	Working range: 5–75 μM
		Glucose	Working range: 5–75 μM
		Skin temperature	Working range: 25–35 °C
2022	Nanoporous carbon-MXene heterostructured nanocomposite-based epidermal patch [60]	Glucose	Linear range: 3 μM–1.5 mM (R^2^ = 0.9956, sensitivity: 82.68 μA mM^−1^ cm^−2^)
		pH	Linear range: 2–10 (R^2^ = 0.9951)
		Sweat temperature	Linear range: 25–45 °C (R^2^ = 0.9991, sensitivity: 0.921% °C^−1^
		ECG	SNR: 27 ± 5 dB SNR with sweat: 26 ± 9 dB
		EEG	Power spectral density: 0.029 μV^2^/Hz
		EMG	SNR: 21 ± 4 dB SNR with sweat: 19 ± 9 dB
2022	Wearable biosensors based on stretchable fiber-based triboelectric nanogenerators (F-TENG) [61]	Glucose	Linear range: 0–56 mM (R^2^ > 0.997)
		Creatinine	Linear range: 0–88 mM (R^2^ > 0.996)
		Lactate acid	Linear range: 0–200 mM (R^2^ > 0.996)
		Movement	Feasibility validation during exercise
2023	Multifunctional wearable system for mapping body temperature and analyzing sweat [62]	Sweat loss	Feasibility validation during exercise
		Chloride	Linear range: 25–100 mM
		Skin temperature	Feasibility validation during exercise
2024	Multifunctional conductive hydrogel film for wearable sensors [63]	Glucose	Linear range: 20 μM^−1^ mM (R^2^ = 0.99067, sensitivity: 117.6 μA mM^−1^ cm^−2^) Limit of detection: 2.82 μM
		Movement	Feasibility validation during exercise
2024	Multifunctional wearable sensor ACBt/P(AA-MA) hydrogel with an organic–inorganic network [64]	NaCl	Working range: 0.25–5 mg/mL
		pH	Working range: 4.0–8.0
		Temperature	Working range: 20.0–60.6 °C
		Movement	Feasibility validation
		Handwritten	Feasibility validation for digit recognition
2024	Physicochemical-sensing electronic skin for stress response monitoring [65]	Glucose	Sensitivity: 33.65 nA μM^−1^ cm^−2^
		Lactate	Sensitivity: 185.56 nA m M^−1^ cm^−2^
		Uric acid	Sensitivity: 26.36 nA μM^−1^ cm^−2^
		Na^+^	Sensitivity: 58.9 mV/dec
		K^+^	Sensitivity: 60.6 mV/dec
		Ammonium	Sensitivity: 61.2 mV/dec
		Pulse waveform	Pressure sensor linear range: 0–500 Pa (sensitivity: 113.1% kPa^−1^)
		EDA	Feasibility validation during exercise
		Skin temperature	Linear range: 25–50 °C (sensitivity: 0.115% °C^−1^)
2024	Nanofiber membrane-based bionic skin for health management [66]	Glucose	Linear range: 50–500 μM (sensitivity: 3.6 nA μM^−1^)
		Lactic acid	Linear range: 5–25 mM (sensitivity: 156.6 nA mM^−1^)
		pH	Linear range: 3.7–8.5
		Skin temperature	Linear range: 26–46 °C (sensitivity: 0.11% °C^−1^)
		Skin impedance	Feasibility validation during exercise
		EMG	SNR: 10.7 dB

**Table 4:** Summary of the advantages, challenges, and future directions of multi-modal monitoring technologies.

Category	Specific Type	Advantages	Challenges	Future Directions
Sensing Mechanisms	Biochemical Sensing	High specificity High sensitivity	Insufficient stability Limited enzyme lifespan	Develop more stable biochemical recognition units and non-enzyme strategies Establish multi-modal combination mechanisms that enhance signal robustness and physiological relevance
	Physical Sensing	Simple structures Portable Non-contact	Physical environmental interference Nanostructure preparation	
	Biochemical-Physical Coupling	Accuracy	Long-term reliability	
Platform Integration	Monolithic Integrated Platforms	High integration and flexibility	Costs Limited reusability	Promote the development of highly integrated, low-power, and flexible/stretchable platforms Design system-level solutions that are repeatable and scalable for mass production and standardized processes Optimize packaging and anti-contamination strategies to improve long-term reliability
	Modular Combination Platforms	Functional scalability Lower costs Easy maintenance	Interface stability Conformal	
Data-Processing Pattern	Data Calibration	Accuracy, reliability, and less errors Suppressed mechanical artifacts and electrical noise	Less reliance on semi-empirical calibration models Capture complex cross-calibration relationships	Achieve calibration and generalization using large-scale multi-center clinical data Enhance physiological interpretation through AI model analysis, supporting personalized and predictive healthcare
	Data Prediction	Higher prediction sensitivity Intelligent inference and individual assessment Boosted robustness Noise and artifact suppression	Sample size and data quality Model fitting and black-box interpretability Limited un-supervised/semi-supervised use in sweat sensing	
	Data Fusion	Comprehensive physiological/pathological perspective Supported 3PM	Heterogeneous signal fusion Feature-level fusion Vulnerable multi-modal data	

**Table 5:** Design rules for different technical routes (note: more stars (“*”) indicate higher requirements for that performance metric. “*” denotes a low requirement, “**” a moderate requirement, “***” a high requirement, and “****” a very high/critical requirement.).

Purpose	Accuracy	Cost	Response Speed	Comfort	Stability	Operational Complexity
Clinical Application	****	****	***	**	***	****
Sports Monitoring	**	***	***	****	****	**
Daily Assistance	*	*	*	****	***	*

## Data Availability

Not applicable.
